# Validation of ICD codes for the identification of patients with functional seizures

**DOI:** 10.1016/j.seizure.2025.03.002

**Published:** 2025-03-05

**Authors:** Jonah Fox, Hannah E. Branstetter, Robert Havranek, Murli Mishra, Nicholas S. Mallett

**Affiliations:** Department of Neurology, Vanderbilt University Medical Center, Nashville, TN, United States

**Keywords:** Psychogenic non-epileptic seizures, Sensitivity, Specificity, Administrative database, Diagnosis codes

## Abstract

**Objective:**

To evaluate the validity of ICD-9-CM and ICD-10 codes for the identification of patients with functional seizures (FS).

**Methods:**

We evaluated the charts of 800 patients including 400 in an institution wide sample and 400 in an epilepsy monitoring unit (EMU) sample. Half of the patients from each sample came from 2012–2013 and 2022–2023 since ICD-9-CM codes and ICD-10 codes were exclusively used in these respective periods. The charts of each patient were manually reviewed and evaluated for the presence of epilepsy and FS. Based on the 2013 International League Against Epilepsy (ILAE) Nonepileptic Seizures Task Force guidelines we determined whether a patient had either presumptive (clinically established or documented) or probable/possible FS. We evaluated ICD-9-CM codes 300.11 (conversion disorder) and 780.39 (other convulsions) as well as ICD-10 codes F44.5 (conversion disorder or functional neurological disorder with seizures or convulsions) and R56.9 (unspecified convulsions). The positive predictive value (PPV) of each ICD code was calculated in the institution wide sample and the sensitivity, specificity, PPV and negative predictive values (NPV) were calculated in the EMU sample.

**Results:**

In the institution wide sample, F44.5 had a PPV of 74.0 % (64.6–81.6) for presumptive FS and 80.0 % (71.1–86.7) when probable/possible FS patients were included. The code 300.11 had a PPV of 52.0 % (42.3–61.5) for presumptive FS and 59.0 % (49.2–68.1) when probable/possible FS patients were included. Codes R56.9 and 780.39 had PPVs that were equal to or less than 20 %. In the EMU sample, the code F44.5 had a sensitivity, specificity, PPV and NPV of 67.1 % (56.3–76.3), 95.8 % (90.5–98.2), 91.7 % (81.9–96.4), and 80.7 % (73.4–86.4), respectively for presumptive FS. The code 300.11 had a sensitivity, specificity, PPV and NPV of 30.1 % (20.8–41.4), 95.3 % (90.1–97.8), 78.6 % (60.5–89.8), and 70.4 % (63.1–76.7), respectively for presumptive FS. The codes R56.9 and 780.39 performed poorly.

**Significance:**

ICD codes had a mixed performance when used to identify patients with FS. ICD-10 code F44.5 appeared to perform the best overall.

## Introduction

Functional seizures (FS), also called psychogenic non-epileptic seizures (PNES), present as episodes of time-limited motor, sensory, autonomic, or cognitive signs and symptoms that superficially resemble epileptic seizures. Unlike epilepsy, FS are not caused by ictal epileptiform activity but rather are thought to be due to a complex interaction of biopsychosocial factors. Studies have shown that, of patients presenting to tertiary care epilepsy monitoring units, 20–40 % of adults and 10–23 % of children presenting with “drug resistant seizures” were found to have FS [1].

The epidemiology of FS is not well understood since it is understudied, and there is often a long delay from onset of symptoms to diagnosis as well as frequent misdiagnosis [2]. The incidence and prevalence of FS vary widely between studies. One study using data from 1992 to 1996 in Iceland showed an estimated incidence of 1.4 cases per 100,000 people per year [3]. A retrospective study done using data from 1995 to 1998 for a county in Ohio, USA showed an estimated incidence of up to 4.6 cases per 100,000 people per year [4]. Prevalence estimates for FS also have a wide range. A study from 2000 used available and estimated data from patients referred to epilepsy centers to calculate an estimated prevalence of FS in the general population of 2–33 per 100, 000 persons [5]. A more recent systematic review and meta-analysis from 2021 calculated an estimated incidence of 3.1 per 100,000 population per year using data from multiple countries, and the author applied a formula to calculate an estimated prevalence of 108.5 per 100, 000 population for the year 2019 in the USA [6]. A systematic review and meta-analysis of observational studies which reported on the dual diagnosis of epilepsy and FS found that patients with FS had a mean co-occurring epilepsy frequency of 22 % (range of 0–90 %) and patients with epilepsy had a mean co-occurring FS frequency of 12 % (range of 1–62 %) [7]. FS are associated with significant morbidity and mortality, high medical costs, and disability; therefore, more research is needed to characterize the epidemiology of FS to improve care and inform policy decisions [8].

Large administrative claims datasets that use diagnosis codes such as ICD-9-CM or ICD-10 are one of the most powerful tools for population-based research. They can be used to estimate disease incidence and prevalence, perform epidemiological studies, and for disease surveillance [9]. In addition, ICD codes can be used to track real-world outcomes on a population-level via analysis of electronic health records and claims. There is a large volume of epilepsy research that relies on this method but not as much for FS since the relevant ICD codes have not been systematically validated [10–12]. ICD codes perform with different degrees of accuracy for identifying particular conditions. For example, studies on the sensitivity and positive predictive value of ICD codes for conditions such as acute stroke (sensitivity ≥82 %, PPV ≤68 %), opioid overdose (81 % PPV), and uveitis (PPV 61 % with range 0–100 %) have large ranges and variable accuracy [13–15].

ICD codes perform relatively well for population-based epilepsy research [9,16–18]. The incidence and prevalence of epilepsy has been previously evaluated using validated ICD codes in electronic health records and claims data [19,20]. These studies utilized operational definitions of epilepsy that placed greater weight on more specific epilepsy ICD codes such as G40.* compared to R56.* and also took into account anti-seizure medication prescription data.

In order to perform population-based research on patients with FS, it is essential to know whether ICD codes can be used to accurately and sensitively identify patients with clinically diagnosed FS. This is particularly relevant for FS given that misdiagnosis and underdiagnosis is common. There have been limited studies validating the use of ICD-9-CM and ICD-10 codes for the identification of patients with FS. Thus, our aim was to assess their performance at a large tertiary academic medical center in the United States.

## Methods

### Study design

This cross-sectional study was performed at a large tertiary medical center in the United States with a catchment area that primarily includes Tennessee, north Alabama, south Kentucky. Adult and pediatric patients were included. Data was collected on patients who received care at our medical center between 2012–2013 and 2022–2023. This was done so that we could independently evaluate the performance of ICD-9-CM and ICD-10 codes during periods of time that each coding system was exclusively utilized. At our center ICD-9-CM and ICD-10 codes were exclusively used between 2012–2013 and 2022–2023, respectively. We evaluated ICD-9-CM codes 780.39 (other convulsions) and 300.11 (conversion disorder) and ICD-10 codes F44.5 (conversion disorder or functional neurological disorder with seizures or convulsions) and R56.9 (unspecified convulsions). This study was reviewed and approved by the Vanderbilt University Medical Center Institutional Review Board. Informed consent was waived because the study data was collected retrospectively.

### Data source

Validation of ICD codes would ideally be performed on a random sample of all patients in the hospital system. Given that FS is not a common diagnosis it would not be practical to obtain a random sample of all patients in the hospital since it would require a review of many thousands of charts. Therefore, we obtained one random sample from all hospitalized patients in our epilepsy monitoring unit (EMU) and another sample that was obtained with an ICD code search on patients seen within our health system. One random sample of 200 patients was obtained of patients admitted to our EMU for each period (2012–2013 and 2022–2023). Strengths of the EMU sample include a high level of diagnostic certainty and the ability to calculate sensitivities, specificities, positive predictive value (PPV), and negative predictive value (NPV). However, limitations with this sample include that there could be a sampling bias given that not all types of patients are referred to the EMU. Therefore, additional samples were obtained on 100 patients who had a medical encounter in any setting between 2012 and 2013 with ICD-9-CM code 780.39 and another sample of 100 patients with ICD-9-CM code 300.11. For the period between 2022 and 2023, 100 patients were sampled with ICD-10 code F44.5 and an additional 100 patients with ICD-10 code R56.9. The limitation of this sample is that we would only be able to report on the PPV and that there could be a lower level of diagnostic certainty. The search was performed on the Vanderbilt Research Derivative which is a searchable database of millions of electronic health records from patients seen at our institution. The ICD codes were evaluated for only during the specific timeframes for the respective ICD-9 and ICD-10 cohorts (i.e., 2012–2013 and 2022–2023). We did not exclude patients from the institutional sample if they happened to have EMU testing however, we excluded duplicate patients to avoid double counting particular individuals.

### Chart review and diagnostic criteria

Chart review was performed by neurology residents (NM, HB, RH and MM) and epileptologist (JF). The charts were approximately equally divided between the neurology residents and epileptologist. However, after the neurology residents were finished the epileptologist reviewed their data entries for accuracy. Chart reviewers were blinded to patient ICD codes throughout the data collection process including during the epileptologist’s review. The diagnosis of FS was based on the 2013 International League Against Epilepsy (ILAE) Nonepileptic Seizures Task Force guidelines [21]. Patients were considered to have presumptive (documented or clinically established) FS if no epileptiform activity was observed during a habitual event recorded on video EEG that had semiology for which an ictal discharge would be expected if the event was an epileptic seizure or the event had typical FS semiology. The patient was considered to have probable or possible FS if the patient had an EEG that did not detect epileptiform activity and there was documentation in the chart that a clinician observed an event (including by video) with a semiology highly suggestive of FS or there was a detailed description of such an event obtained from clinical history. A patient was considered to have definite epilepsy if a habitual event was recorded on video EEG that was associated with epileptiform activity and a history consistent with epilepsy according to ILAE criteria [22]. The patient could also be considered to have probable epilepsy if they had an EEG with interictal epileptiform activity, an observed or described event that was suggestive of an epileptic seizure, and a history suggestive of epilepsy documented in the chart. The classifications of FS and epilepsy were not considered to be mutually exclusive, and patients could be classified as having both conditions with their respective diagnostic certainties. The authors recognized that the requirement of an abnormal EEG may have excluded some patients given that a proportion of those with epilepsy may not have interictal epileptiform discharges however it is not rare for even experienced epileptologists to occasionally misdiagnose patients based on clinical history alone. The patient could also be considered to have types of non-epileptic events other than FS such as syncope, panic attacks, or tics. Regardless of whether the patient was in the EMU or not, all EEG reports for each patient were reviewed as well as relevant notes and other documentation in the chart.

### Variables and data collection

The following variables were extracted from the record for each patient: date of birth, sex, the presence of interictal epileptiform discharges, if a habitual event was recorded, and if so, did the event have an EEG correlate. A determination was made if the patient had presumptive FS, definite epilepsy, probable/possible FS, probable epilepsy, and non-epileptic events other than FS. A patient could not be defined as having both definite and probable epilepsy or presumptive and probable/possible FS, but all other combinations of designations were permitted. In the EMU samples, the number of EMU admissions, date of first EMU admission and collective duration of all EMU admissions were recorded. In the institution wide sample, the date of first neurology contact, types of hospital encounters (emergency department, inpatient and EMU) as well as the duration of longest EEG were recorded.

### Statistical analysis

The sensitivity, specificity, PPV, and NPV with 95 % confidence intervals were calculated for each ICD-9-CM and ICD-10 code in the respective EMU samples. The hybrid Wilson/Brown method was used to calculate confidence intervals. In the institution wide sample, we only calculated the PPVs. We provided two sets of calculations with one counting only presumptive FS cases and a second set where probable/possible FS was also counted. In addition, we reported on the patients who had either co-occurring epilepsy, only epilepsy, non-epileptic events other than FS, and non-diagnostic or unknown diagnoses. A Chi-square test was used to compare the PPV of the pediatric and adult population groups. All analyses were performed using GraphPad Prism 10.4.1 (GraphPad Software, San Diego, California).

## 3. Results

### 3.1. Sensitivity, specificity, PPV and NPV in the EMU sample

The charts of 400 patients were reviewed and 60.5 % (242/400) were female (Table1). At the time of the first EMU admission the average patient age was 27.2 years (standard deviation, SD = 19.6). The proportion of patients that were less than 18 years of age was 41.5 % (166/400). The majority or 78.3 % (313/400) had only one EMU admission, 12.5 % (50/400) had 2 EMU admissions, and the remaining 8.8 % (35/400) had 3 or more EMU admissions. 75.6 % (303/400) had a typical event recorded during any EMU admission. A diagnosis of presumptive FS was made in 39.3 % (157/400) of patients and an additional 3.8 % (15/400) had probable/possible FS. A diagnosis of definite epilepsy was made in 36.8 % (147/400) of patients and an additional 6.3 % (25/400) had probable epilepsy. Co-occurring presumptive FS and definite epilepsy was present in 5.3 % (21/400) of patients. In the 2022–2023 EMU sample, there were 8 patients with co-occurring presumptive FS and definite epilepsy. 62.5 % (5/8) of these patients were coded with F44.5, 75.0 % (6/8) were coded with a G40.* code, and 37.5 % (3/8) had both. An additional 5.6 % (22/400) patients had a diagnosis of non-epileptic events that were not FS such as motor tics and sleep myoclonus. A total of 17.5 % (70/400) of patients in the EMU group had admissions that were non-diagnostic only. From the 2022 to 2023 group, 30.0 % (60/200) had a diagnosis code of F44.5 and 96.5 % (193/200) had a diagnosis code of R56.9. Among the 2012–2013 group, 14.0 % (28/200) had a diagnosis code of 300.11 and 92.5 % (185/200) had a diagnosis code of 780.39. 15.1 % (28/185) of patients with ICD code 780.39 were also coded with 300.11. Whereas all (28/28) of the patients who had ICD code 300.11 also had 780.39. 31.1 % (60/193) of patients who had ICD code R56.9 also were coded with F44.5. All (60/60) patients who had ICD code F44.5 also had R56.9.

The ICD-10 code F44.5 had a sensitivity of 67.1 %, specificity of 95.8 %, PPV of 91.7 % and NPV of 80.7 % when only counting patients with presumptive FS (Table 2). When the 3 patients with probable/possible FS were included, the performance changed little except for the PPV increasing to 96.7 %. A small minority of 1.7 % (1/60) of patients with F44.5 had a diagnosis of definite epilepsy without co-occurring FS (presumptive or probable/possible). The ICD-10 code R56.9 had a sensitivity of 100.0 %, specificity of 0.1 %, PPV of 42.5 % and NPV of 1.0 % when only counting patients with presumptive FS. The PPV increased to 45.1 % when 5 patients with probable/possible FS were included. 28.0 % (54/193) of patients with R56.9 had a diagnosis of definite epilepsy without co-occurring FS (presumptive or probable/possible).

The ICD-9 code 300.11 had a sensitivity of 30.1 %, specificity of 95.3 %, PPV of 78.6 % and NPV of 70.4 % for detection of patients with presumptive FS only (Table 3). When the 3 patients with probable/possible FS were included the PPV increased to 89.3 %. 7.1 % of patients with 300.11 had a diagnosis of definite epilepsy without co-occurring FS (presumptive or probable/possible). The ICD-9 code 780.39 had a sensitivity of 97.3 %, specificity of 10.2 %, PPV of 38.4 % and NPV of 86.7 % for detection of patients with presumptive FS only. When the 7 patients with possible or probable FS were included the PPV increased to 42.2 %. 33.0 % (61/185) of patients with 780.39 had a diagnosis of definite epilepsy without co-occurring FS (presumptive or probable/possible).

Among patients in the EMU sample there were 2 patients with F44.5 that were not classified as having FS. 1 had definite epilepsy and the other had a non-diagnostic admission. The non-diagnostic admission had insufficient information to make a determination of probable/possible FS. There were also 3 patients with 300.11 that were not classified as having FS. 2 had definite epilepsy and 1 had a non-diagnostic admission with insufficient information to make a determination of probable/possible FS.

### 3.2. PPV in the institution wide sample

The charts of a total of 400 patients were reviewed and 63.3 % (253/400) were female. The mean age at the time of first encounter with neurology was 32.4 years (SD = 54.0). A total of 11.8 % (47/400) patients did not have any encounter with neurology at our institution. Pediatric patients constituted 37.1 % (131/353) of patients with neurology encounters. Among all patients, 81.3 % (325/400) had any outpatient encounter, 78.8 % (315/400) had an emergency department encounter, 46.3 % (185/400) had an inpatient encounter (excluding EMU), and 39.8 % (159/400) had an EMU admission. A total of 79.3 % (317/400) had an EEG of any length. The most common length of the longest single EEG recording was greater than 24 h in 48.3 % (193/400) of patients, 12–24 h in 3.8 % (15/400) of patients, 2–12 h in 11.0 % (44/400) of patients, and less than 2 h in 16.3 % (65/400) of patients. A typical event was recorded in 63.1 % (200/317) of patients who had EEG. A diagnosis of presumptive FS was made in 39.5 % (158/400) of patients and an additional 4.3 % (17/400) had probable/possible FS. A diagnosis of definite epilepsy was made in 11.5 % (46/400) of patients and an additional 5.8 % (23/400) had probable epilepsy. Co-occurring presumptive FS and epilepsy was present in 3.0 % (12/400) of patients. A further 2.0 % (8/400) patients had a diagnosis of non-epileptic events other than FS such as behavioral outbursts or panic attacks. A total of 41.5 % (166/400) had an unclear diagnosis.

The PPV of F44.5 was 74.0 % for presumptive FS only and 80.0 % for presumptive or probable/possible FS (Table 4). The PPV of R56.9 for presumptive FS only was 14.0 % and 16.0 % for presumptive or probable/possible FS. 2 % (2/100) of patients with diagnosis code F44.5 had confirmed epilepsy without co-occurring FS (presumptive or probable/possible). 17 % (17/100) of patients with diagnosis code R56.9 had confirmed epilepsy without co-occurring FS (presumptive or probable/possible). The PPV of 300.11 was 52.0 % for presumptive FS only and 59.0 % for presumptive or probable/possible FS (Table 5). The PPV of 780.39 was 18.0 % for presumptive FS only and 20.0 % for presumptive or probable/possible FS. 2 % (2/100) of patients with diagnosis code 300.11 had confirmed epilepsy without co-occurring FS (presumptive or probable/possible). 12 % (17/100) of patients with diagnosis code 780.39 had confirmed epilepsy without co-occurring FS (presumptive or probable/possible). We compared the PPV for presumptive or probable/possible between pediatric and adult populations but did not find that there was a statistically significant difference between the groups. There was a trend towards a higher PPV for F44.5 in the adult population but it did not reach statistical significance (*p* = 0.09) (Table 6).

There were 20 patients with F44.5 that were not classified as having FS. 2 of these patients had definite epilepsy and 2 had probable/possible epilepsy. The other 18 had insufficient information or no EEG such that they could not be classified as having probable/possible FS. There were also 41 patients with 300.11 that were not classified as having FS. There were 2 who had non-epileptic events that were not FS (torticollis and behavioral outbursts), 2 had confirmed epilepsy, and 37 had either insufficient clinical information or no EEG.

## Discussion

The ICD codes utilized for this study had varying accuracy in identifying FS. The ICD codes R56.9 and 780.39 were very nonspecific and included a non-insignificant proportion of patients who had epilepsy without coexisting FS. This should not be surprising given that these codes are for ‘unspecified convulsions’. ICD-10 code F44.5 appeared to be the most useful overall with a good PPV for both the EMU and non-EMU groups as well as a good specificity and modest sensitivity in the EMU group. The ICD-9 code 300.11 had a good specificity and PPV in the EMU group, but this did not translate to its PPV in the non-EMU group. We suspect that this is in part because many EMU admissions have a relatively high pre-test probability for FS since a large proportion of patients who are referred to the EMU have FS, but this is less true elsewhere in the hospital [23]. The sensitivity of 300.11 was also relatively poor. F44.5 may have performed better than 300.11 in part because there was a 10-year gap between the time periods that they were evaluated. Awareness and diagnosis of FS has likely improved to some extent during this time. In addition, F44.5 may have also performed better because it codes for *conversion disorder or functional neurological disorder with seizures or convulsions* whereas 300.11 is less specific since it codes for the more broad *conversion disorder*.

There are relatively few previous studies that evaluated the accuracy of ICD codes specifically for the diagnosis of FS or even functional neurological disorders more generally. We were able to find one study which evaluated the PPV of ICD-10 code F44.5 in the Veterans Affairs Electronic Medical Record in Connecticut [24]. Though this code was the best performing in our study overall, it had a lower PPV in this VA cohort with an average of 44 % and a range in the included medical centers of 14–65 %. The discussion of this article postulated that the reason for such a low PPV may be related to the lookup diagnosis function when entering an ICD code in the VA electronic medical record. Therefore, there may be differences among the lookup diagnosis functions between electronic medical records systems that influence coding. It is also possible that since our study was performed at an academic medical center that providers and coders here were more familiar with FS and therefore were more likely to use the most appropriate ICD code. In another study out of Norway cases were identified by using ICD codes F44.5 and R56.8, and the authors of the study reviewed each patient’s case and confirmed that the patients met criteria for FS based on the ILAE 2013 criteria [25]. Of the confirmed cases, only 24 % had the more specific diagnosis code of F44.5 while the rest had the less specific diagnosis code R56.8.

There are likely multiple factors which influence the accuracy of ICD codes for FS. One reason is related to the fact that FS is frequently misdiagnosed, and that non-neurologist knowledge of the condition is relatively poor. For example, a systematic review found that many health care providers had an inaccurate understanding of FS as well as uncertainty regarding the diagnosis and treatment of the condition [26]. This is likely exacerbated by the various medical terms that have been used to describe FS over the years [27]. It has also been shown that physicians often use ambiguous language and engage in “diagnostic hedging” when documenting FS despite the existence of an ILAE 2013 diagnostic criteria. This is thought to be due to fear of medicolegal implications and decreased billing capability for “psychiatric” ICD codes as compared to “medical” ICD codes [28]. It should be noted that this is a misconception since most insurers in the United States including Medicare and Medicaid reimburse for both EEGs and clinical care equally for FS as they do for epilepsy [29]. It is also suspected that professional coders may not appreciate the distinction between FS and epilepsy and assign the incorrect code.

The accuracy of diagnosis codes for FS is important because this is commonly how patients are identified in research including epidemiological studies, as well as evaluations of population-level outcomes, healthcare resource utilization, cost analyses, and more [30,31]. Improper coding of FS patients may introduce inaccuracies and result in an underestimation of FS incidence and prevalence in the population. This may result in the misallocation and underfunding of FS research and ultimately lead to poorer outcomes for patients. Improved ICD coding for FS could potentially be achieved through enhanced provider and coder education about the distinctions between FS and epilepsy. In addition, providers should be better educated regarding the ILAE 2013 diagnostic criteria for FS which may lead to reduced diagnostic hedging. F44.5 is currently reimbursable by Medicare and Medicaid for EEG and clinical care therefore providers should not be averse to using it [29]. Providers and coders should attempt to code as accurately as possible and consider that in some instances multiple codes may be appropriate. The non-specific codes such as R56.9 should be avoided if a specific diagnosis has been made. In addition, if a patient has co-occurring FS and epilepsy they should appropriately be coded with both F44.5 and G40.*.

Some studies have used more complex algorithms for identifying patients with FS. For example, Goleva et al. utilized an automated phenotyping algorithm that used a combination of ICD codes, CPT codes and natural language processing [32]. Potential limitations of using a more sophisticated algorithm like this is that it may need to be revali-dated before it can be used at other institutions and the software required for running it may not be universally available like ICD codes. However, for individual studies it may be the best method for identifying patients with FS at this point in time.

The results of this study need to be interpreted in the context of its limitations. This study was performed at a single center using data from 2012–2013 and 2022–2023. For these results to be more generalizable and have larger power, it would be helpful if future studies were multicenter and analyzed over different timepoints. As mentioned above, different centers have different electronic medical records which may affect the way that ICD codes are entered into the chart and affect the results of the study. In addition, providers and coders at different centers may be more or less likely to use particular codes for patients with FS for the reasons described previously. The relative proportion of patients that have FS compared to epilepsy and other diagnoses may also differ between institutions. Additionally, our center has epilepsy specialists and 24-h EEG availability which may also have affected our results. This study focused on ICD-9 and ICD-10 codes, but future studies should include ICD-11 codes as they become more widely used over time. A future study could take a different approach and instead of deciding which ICD codes to evaluate prior to data collection could alternatively examine a group of patients with FS and see which ICD codes they are given. It would also be of interest to evaluate how the presence of a particular code impacts future coder behavior. For example, if a patient receives the code F44.5 at one point in time does it have any impact on which codes are subsequently used. In addition, ICD codes should be evaluated for the purpose of identifying patients with co-occurring epilepsy and FS.

## Conclusion

We found that F44.5 overall performed well for the identification of patients with FS but had a limited sensitivity. Other diagnosis codes performed relatively poorly and probably should not be used in epidemiological research unless they are supplemented by other methods to confirm the patient’s diagnosis. Efforts should be made to improve the accuracy of medical coding for FS which may ultimately contribute to the betterment of both our understanding and treatment of this condition.

## Figures and Tables

**Table 1:** Summary of study sample demographic and clinical characteristics.

	Total sample (*n* = 800)	
	EMU sample (*n* = 400)	Institutional sample (*n* = 400)
Female (%)	242 (60.5 %)	253 (63.3 %)
Mean age in years (SD)	27.2 (19.6)	32.4 (54.0)
Proportion of pediatric patients (%)	166 (41.5 %)	131 (37.1 %)
EEG testing performed (%)	400 (100 %)	317 (79.3 %)
EMU testing performed (%)	400 (100 %)	159 (39.8 %)
Typical event recorded (%)	303 (75.6 %)	200 (50.0 %)
Presumptive FS (%)	157 (39.3 %)	158 (39.5 %)
Probable/possible FS (%)	15 (3.8 %)	17 (4.3 %)
Definite epilepsy (%)	147 (36.8 %)	46 (11.5 %)
Probable/possible epilepsy (%)	25 (6.3 %)	23 (5.8 %)
Co-occurring presumptive FS and definite epilepsy (%)	21 (5.3 %)	12 (3.0 %)
Diagnosis of non-epileptic events other than FS (%)	22 (5.6 %)	8 (2.0 %)
Undetermined diagnosis (%)	70 (17.5 %)	166 (41.5 %)
Non-diagnostic EMU (%)	70 (17.5 %)	14 (8.8 %)

**Table 2:** Performance of ICD-10 codes F44.5 and R56.9 for detection of patients with FS in the EMU.

	F44.5 Presumptive FS only	F44.5 Presumptive FS or probable/possible FS	R56.9 Presumptive FS only	R56.9 Presumptive FS or probable/possible FS
Sensitivity % (95 % CI) – raw numbers	67.1 (56.3–76.3) – 55/82	66.7 (56.2–75.7) – 58/87	100 (95.5–1.0) – 82/82	100 (95.8–1.0) – 87/87
Specificity % (95 % CI) – raw numbers	95.8 (90.5–98.2) – 113/118	98.2 (93.8–99.7) – 111/113	0.1 (0.03–0.12) – 1/118	0.1 (0.03–12.2) – 1/113
Positive predictive value % (95 % CI) – raw numbers	91.7 (81.9–96.4) – 55/60	96.7 (88.6–99.4) – 58/60	42.5 (35.7–49.5) – 82/193	45.1 (38.2–52.1) – 87/193
Negative predictive value % (95 % CI) – raw numbers	80.7 (73.4–86.4) – 113/140	79.3 (71.8–85.2) – 111/140	1.0 (64.6–1.0) – 7/7	1.0 (64.6–1.0) – 7/7

FS: functional seizures, CI: confidence interval.

**Table 3:** Performance of ICD-9 codes 300.11 and 780.39 for detection of patients with FS in the EMU.

	300.11 Presumptive FS only	300.11 Presumptive FS or probable/possible FS	780.39 Presumptive FS only	780.39 Presumptive FS or probable/possible FS
Sensitivity % (95 % CI) – raw numbers	30.1 (20.8–41.4) – 22/73	30.9 (21.9–41.6) – 25/81	97.3 (90.6–99.5) – 71/73	96.3 (89.7–99.0) – 78/81
Specificity % (95 % CI) – raw numbers	95.3 (90.1–97.8) – 121/127	97.5 (92.9–99.3) – 116/119	10.2 (6.1–16.7) – 12/127	10.1 (5.9–16.8) – 12/119
Positive predictive value % (95 % CI) – raw numbers	78.6 (60.5–89.8) – 22/28	89.3 (72.8–96.3) – 25/28	38.4 (31.7–45.6) – 71/185	42.2 (35.3–49.4) – 78/185
Negative predictive value % (95 % CI) – raw numbers	70.4 (63.1–76.7) – 121/172	67.4 (60.1–74.0) – 116/172	86.7 (62.1–97.6) – 13/15	80.0 (54.8–93.0) – 12/15

FS: functional seizures, CI: confidence interval.

**Table 4:** Positive predictive value of ICD-10 codes F44.5 and R56.9 for detection of patients with FS in the institutional sample.

	F44.5 Presumptive FS only	F44.5 Presumptive FS or probable/possible FS	R56.9 Presumptive FS only	R56.9 Presumptive FS or probable/possible FS
Positive predictive value % (95 % CI) – raw numbers	74.0 (64.6–81.6) – 74/100	80.0 (71.1–86.7) – 80/100	14.0 (8.53–22.1) – 14/100	16.0 (10.1–24.4) – 16/100

FS: functional seizures, CI: confidence interval.

**Table 5:** Positive predictive value of ICD-9 codes 300.11 and 780.39 for detection of patients with FS in the institutional sample.

	300.11 Presumptive FS only	300.11 Presumptive FS or probable/possible FS	780.39 Presumptive FS only	780.39 Presumptive FS or probable/possible FS
Positive predictive value % (95 % CI) – raw numbers	52.0 (42.3–61.5) – 52/100	59.0 (49.2–68.1) – 59/100	18.0 (11.7–26.7) – 18/100	20.0 (13.3–28.9) – 20/100

FS: functional seizures, CI: confidence interval.

**Table 6:** The PPV of ICD-9 and ICD-10 codes for detection of patients with FS compared between pediatric and adult populations among patients with any type of neurology encounter in the institutional sample.

	Pediatric population Positive predictive value % (95 % CI) – raw numbers	Adult population Positive predictive value % (95 % CI) – raw numbers	*P* value
F44.5	75.9 (57.9–87.8) – 22/29	89.2 (79.4–94.7) – 58/65	0.09
R56.9	16.7 (8.3–30.6) – 7/42	22.0 (12.0–36.7) – 9/41	0.54
300.11	65.4 (46.2–80.1) 17/26	65.6 (53.4–76.1) – 42/64	0.98
780.39	20.6 (10.4–36.8) – 7/34	25.0 (15.2–38.2) – 13/52	0.64

CI: confidence interval.
